# Vegan and Plant-Based Diets in the Management of Metabolic Syndrome: A Narrative Review from Anti-Inflammatory and Antithrombotic Perspectives

**DOI:** 10.3390/nu17162656

**Published:** 2025-08-15

**Authors:** Fatemeh Jafarnezhad, Ata Nazarzadeh, Haniyeh Bazavar, Shayan Keramat, Ireneusz Ryszkiel, Agata Stanek

**Affiliations:** 1 Department of Hematology, Faculty of Medicine, Mashhad University of Medical Sciences, Mashhad 9177948974, Iran; fatemeh.jafarnejad1995@gmail.com (F.J.); atanazar4@gmail.com (A.N.); 2 Department of Hematology, Zanjan University of Medical Science, Senman 4513956184, Iran; hani79.bazavar@gmail.com; 3 VAS-European Independent Foundation in Angiology/Vascular Medicine, Via GB Grassi 74, 20157 Milan, Italy; shayan.sk1993@gmail.com; 4 Support Association of Patients of Burger’s Disease, Burger’s Disease NGO, Mashhad 9183785195, Iran; 5 Department of Internal Medicine, Metabolic Diseases and Angiology, Faculty of Health Sciences in Katowice, Medical University of Silesia, Ziołowa 45/47, 40-635 Katowice, Poland; ryszkielirek@gmail.com

**Keywords:** vegan diet, metabolic syndrome, thrombosis, inflammation, cardiovascular diseases, type 2 diabetes

## Abstract

Metabolic syndrome (MetS) is defined by a combination of metabolic abnormalities, such as central obesity, insulin resistance, hypertension, and dyslipidemia, and significantly increases the risk of cardiovascular diseases and type 2 diabetes. The high prevalence of MetS is a public health concern, necessitating rapid identification and intervention strategies to prevent this emerging epidemic. Diagnosing MetS requires the presence of three or more of these abnormalities, underscoring the need for effective management approaches. Despite a growing body of literature, limited reviews have critically evaluated the complex interplay between metabolic dysfunction, inflammation, and coagulation, particularly in the context of dietary interventions. Therefore, this article reviews the relationship between metabolic syndrome, inflammation, and thrombotic diseases, with an emphasis on their impacts on hematological health. Furthermore, this review explores the potential role of vegetarian and vegan dietary patterns in controlling these processes and improving hematological outcomes. This narrative review aims to critically evaluate current research on the inflammatory and thrombotic implications of MetS and assess the potential modulating role of vegan and plant-based diets within this context.

## 1. Introduction

Metabolic syndrome (MetS) is a cluster of interrelated metabolic abnormalities—such as central obesity, insulin resistance (IR), dyslipidemia, and hypertension—that collectively elevate the risk of type 2 diabetes mellitus (T2DM) and cardiovascular disease (CVD) [1,2]. Furthermore, these qualities amplify the risk of atherosclerosis and other metabolic disorders. Although definitions vary slightly among health authorities, central adiposity and IR are consistently emphasized as central components [3].

The occurrence of MetS is growing in both developed and developing nations [4]. Urbanization, reduced physical activity, and diets high in ultra-processed food significantly contribute to its increasing incidence [5]. It is estimated that almost a quarter of adults in industrialized countries meet the diagnostic criteria, while low- and middle-income countries are experiencing a growing number of cases due to changing lifestyles [6]. Genetic and environmental factors further influence susceptibility, emphasizing the need for targeted interventions [3].

The syndrome’s pathophysiology includes hyperglycemia caused by impaired glucose metabolism and insulin resistance (IR) [2]. Adipose tissue releases inflammatory cytokines, leading to chronic low-grade inflammation that worsens metabolic dysfunction [7]. Furthermore, oxidative stress significantly contributes to endothelial dysfunction, exacerbates hypertension, and promotes atherosclerosis [8]. Therefore, addressing these mechanisms is crucial for effective prevention and treatment strategies [6].

Metabolic syndrome also increases the risk of thrombotic diseases and systemic inflammation, leading to both arterial and venous thrombosis through various mechanisms [9]. Central obesity and insulin resistance are hallmark features of MetS that induce chronic low-grade inflammation, promoting atherothrombosis and cardiovascular pathology [10]. This inflammatory process leads to the development of atherosclerotic plaques and increases the likelihood of thrombotic events [11]. This process leads to plaque formation and destabilization, increasing the probability of acute cardiovascular incidents such as myocardial infarction and stroke [12]. Additionally, the prothrombotic state in MetS involves multiple pathways, including coagulation factor activation and reduced fibrinolysis, as discussed further in Section 3 and Section 4 [13]. This imbalanced coagulation and fibrinolysis increase the risk of arterial and venous thromboembolism, strongly contributing to deep vein thrombosis and pulmonary embolism [14]. In addition to the effects on thrombosis, MetS is tightly linked to chronic inflammation, creating a looped cycle that aggravates metabolic dysfunction and vascular damage [15]. The created inflammatory environment not only accelerates the progression of cardiovascular diseases but also increases the risk of other metabolic complications, such as IR and hepatic steatosis [16].

Considering the complex relations between inflammation, coagulation, and endothelial dysfunction in MetS, therapeutic strategies should target both metabolic control and inflammation in order to reduce thrombotic risks and improve long-term cardiovascular outcomes. Within the broader landscape of dietary interventions, a plant-based diet is a dietary pattern characterized by a low frequency of animal food consumption [17]. This category encompasses vegetarian and vegan diets [17]. Vegetarian diets include dairy and eggs, while a vegan diet does not contain any type of animal-derived food [18]. These diets are recognized for their high fiber content, low saturated fat levels, and richness of phytonutrients, all of which have various health benefits [19]. Studies have shown that plant-based diets have a significant impact on metabolic health and reduce risk factors associated with metabolic syndrome [20]. Vegan and vegetarian diets are associated with improved insulin sensitivity, better glycemic control, and a lower risk of type 2 diabetes [21]. Their high fiber content slows glucose absorption, stabilizes blood sugar, and promotes satiety, aiding in weight management [21]. These diets also improve lipid profiles by lowering LDL, cholesterol, and triglycerides, reducing the risk of atherosclerosis [22]. Plant-based omega-3s support cardiovascular health and lower inflammation [23]. Plant-based diets, rich in polyphenols and omega-3s, offer antioxidant and anti-inflammatory benefits. These compounds reduce oxidative stress and suppress pro-inflammatory pathways, resulting in lower levels of markers such as C-reactive protein (CRP) [24]. Moreover, a diet rich in leafy greens and nuts has been shown to support endothelial function, reduce the risk of clot formation, and promote nitric oxide production, enhancing vascular dilation [25]. Thrombosis—the formation of blood clots that can obstruct blood flow—is closely linked to diet and lifestyle. Lower levels of coagulation factors such as fibrinogen and factor VII, which play critical roles in clot formation, can be observed in individuals following a plant-based diet [26]. Moreover, plant-based omega-3s such as α-linolenic acid (ALA) may reduce thromboxane-mediated platelet aggregation and inflammation [27]. Thus, these dietary patterns may reduce the probability of thrombotic events, such as heart attacks and strokes [25,28].

Despite the mentioned benefits, individuals who are following a plant-based diet should consider certain nutritional deliberations to enhance their health outcomes [29]. Some important nutrients, such as cobalamin (vitamin B12), iron, and long-chain omega-3 fatty acids, are mostly found in animal-derived diets; thus, careful dietary planning and supplementation are required [30]. Inadequate vitamin B12 levels may result in elevated plasma homocysteine concentrations, thereby increasing the risk of cardiovascular complications if not properly addressed [31]. Some studies have indicated that although vegetarians and vegans have lower cholesterol and inflammation rates, they have an increased tendency for platelet aggregation because of lower omega-3 acid intake [32]. These imbalances can be effectively addressed by fortified food and supplement intake, so these individuals can maintain well-adjusted nutrient levels while benefitting from the protective effects of plant-based diets [20,33].

## 2. Metabolic Syndrome

A cluster of metabolic disorders, including central obesity, IR, hypertension, and atherogenic dyslipidemia, is defined as metabolic syndrome [34]. The presence of three or more of the following is known as MetS: a waist circumference of more than 40 inches in men and 35 inches in women; a serum triglyceride level of 150 mg/dL or more; a low-density lipoprotein cholesterol level of less than 40 mg/dL in men or less than 50 mg/dL in women; an increase in fasting glucose of 100 mg/dL or more; and systolic blood pressure values of 130 mmHg or higher or diastolic blood pressure of 85 mmHg or higher [35].

Several factors are involved in the development of MetS, which can include genetic predisposition and multiple environmental or lifestyle factors, including obesity, physical inactivity, and unhealthy eating habits [36].

Recent genetic studies have identified several loci associated with increased susceptibility to MetS [37]. Other loci, including the melanocortin 4 receptor (MC4R), a key regulator of appetite and energy homeostasis; transcription factor 7-like 2 (TCF7L2), involved in glucose metabolism and β-cell function; and adiponectin gene (ADIPOQ), crucial for anti-inflammatory and insulin-sensitizing activities, further support the strong genetic basis of MetS [37]. Genome-wide association studies (GWASs) consistently demonstrate that heritable traits such as obesity, dyslipidemia, and insulin resistance cluster in families, suggesting polygenic inheritance mechanisms [37].

Studies have emphasized the role of genetics in the development of this syndrome. It has been shown that children of obese parents have a higher chance of becoming obese [38]. The mother’s lifestyle and nutrition during pregnancy and after birth can also be involved in the development of MetS [34].

MetS is diagnosed based on physical examination findings and laboratory tests. The classic symptoms of polyuria, polydipsia, and polyphagia indicate the presence of diabetes mellitus as a component of MetS. History and physical examination are critical for the recognition, treatment, and prevention of the disease [39]. Tests may include fasting blood glucose and hemoglobin A1c to screen for insulin resistance and diabetes; a lipid panel to assess for abnormal triglyceride levels, low HDL levels, and high low-density lipoprotein levels; and a basic metabolic panel to assess for kidney dysfunction [20]. Measurement of high-sensitivity C-reactive protein (hs-CRP)—an acute-phase protein produced by the liver in response to systemic inflammation—can also be considered as an optional marker, reflecting the chronic low-grade inflammation characteristic of MetS [40].

The global prevalence of MetS is estimated to be around one quarter of the world’s population, with over a billion people currently affected. Prevalence estimates vary based on the criteria used to define MetS [41]. Urbanization, sedentary lifestyles, and the global spread of Western dietary patterns, rich in saturated fats and refined carbohydrates, have significantly contributed to the rising prevalence of MetS [4].

Recent meta-analyses suggest that in some regions—particularly low- and middle-income countries undergoing rapid economic transition—the prevalence may be increasing at a faster rate than previously predicted [4].

## 3. The Effect of Type 2 Diabetes and Hyperglycemia on Thrombosis and Inflammation

IR happens when normal insulin levels do not work well in fat, muscle, and liver tissues. The pancreas releases more insulin to manage high blood sugar. Over time, this leads to less insulin being produced, resulting in high blood sugar or diabetes [42].

The metabolic milieu of T2DM, characterized by IR, hyperglycemia, and the release of excess free fatty acids, along with other metabolic issues, impacts the vascular wall through a sequence of events such as endothelial dysfunction, platelet hyper-reactivity, oxidative stress, and low-grade inflammation [43]. Hyperglycemia associated with diabetes affects the expression of platelet enzymes and receptors at the megakaryocyte stage. In T2DM, the expression level of the negative platelet regulatory receptor, prostacyclin, is reduced, which increases the platelet response [44]. Mean platelet volume (MPV) can be a marker of platelet activity that can be easily measured. Larger platelets have greater enzymatic and metabolic potential than smaller platelets. Consequently, an increase in MPV can be an indication of platelet activation [45]. Studies show a positive relationship between insulin resistance and MPV [45]. An increase in MPV is seen in patients with higher HbA1c and fasting blood glucose (FBG), indicating hyperglycemia, suggesting that hyperglycemia is associated with increased platelet activity [46]. In a study conducted by Masanori Shimodaira and colleagues in 1876 diabetic patients, it was shown that increased blood sugar causes an increase in MPV [47]. In a 2024 study, Velia Cassano et al. showed that MPV in patients with T2DM is significantly increased, indicating increased platelet reactivity [48].

Platelets obtained from diabetes patients demonstrated higher aggregation rates compared with those from non-diabetic individuals when exposed to low levels of prothrombotic agonists such as collagen, ADP, and thrombin [49,50]. This leads to quicker production of more reactive platelets, resulting in larger, hyper-reactive platelets and increased thrombosis risk [49]. Expression of the platelet surface receptors glycoprotein GP Ib and GP IIb/IIIa is higher in individuals with diabetes [51]. Platelet hyper-reactivity is a critical contributor to the prothrombotic state observed in MetS and T2DM [51]. In addition to increased coagulation activation, patients with MetS and T2DM exhibited abnormal fibrin clot structure characterized by denser, tighter fibrin networks, and reduced fibrinolysis [52]. Antithrombin cofactors—heparan sulfate glycosaminoglycans in the superficial layer of the endothelium—are largely responsible for the anticoagulant properties of the endothelium, and the concentration of these molecules is reduced in the arteries of individuals with T2DM [53]. Chronic inflammation (as associated with T2DM) leads to activation of both the complement system and the kinin–kallikrein system, which activates factor XII and causes increased concentrations of several proteins, including factor VIII, tissue factor, prothrombin, and fibrinogen [54].

Proteins that are increased in diabetes include von Willebrand factor, (pre) kallikrein, factor V, factor VII (activated), factor VIII, factor X, factor XI, prothrombin, and fibrinogen. Concomitant with changes in procoagulant proteins, plasma concentrations of the anticoagulant proteins C and S are reduced in diabetes [29]. Plasma concentrations of some metal ions are altered in diabetes [55]. Total plasma calcium levels have been observed to be increased in individuals with T2DM compared with healthy individuals [54]. In diabetes, zinc concentrations were reduced when compared with healthy individuals [56]. Zinc is essential for endothelial integrity, and its deficiency leads to impaired endothelial protective function and induces a cytokine-mediated inflammatory process [57]. Several studies have implicated zinc as a risk factor for hemorrhagic stroke, particularly in relation to zinc deficiency in obesity and T2DM [53]. Copper levels have been shown to be increased in type 2 diabetes. Copper is an essential cofactor of coagulation factors such as V and VIII, but the effect of copper on coagulation is not well understood [54]. Studies have shown that IR induces local accumulation of inflammatory macrophages. IR produces the chemoattractant protein monocyte chemokine 1 (MCP1), which attracts monocytes and activates pro-inflammatory macrophages. IR also prevents glucose from entering neuronal cells for oxidative phosphorylation, which could explain the potential cause of IR-induced inflammation in terms of macrophage metabolic changes [58].

**Figure 1:**
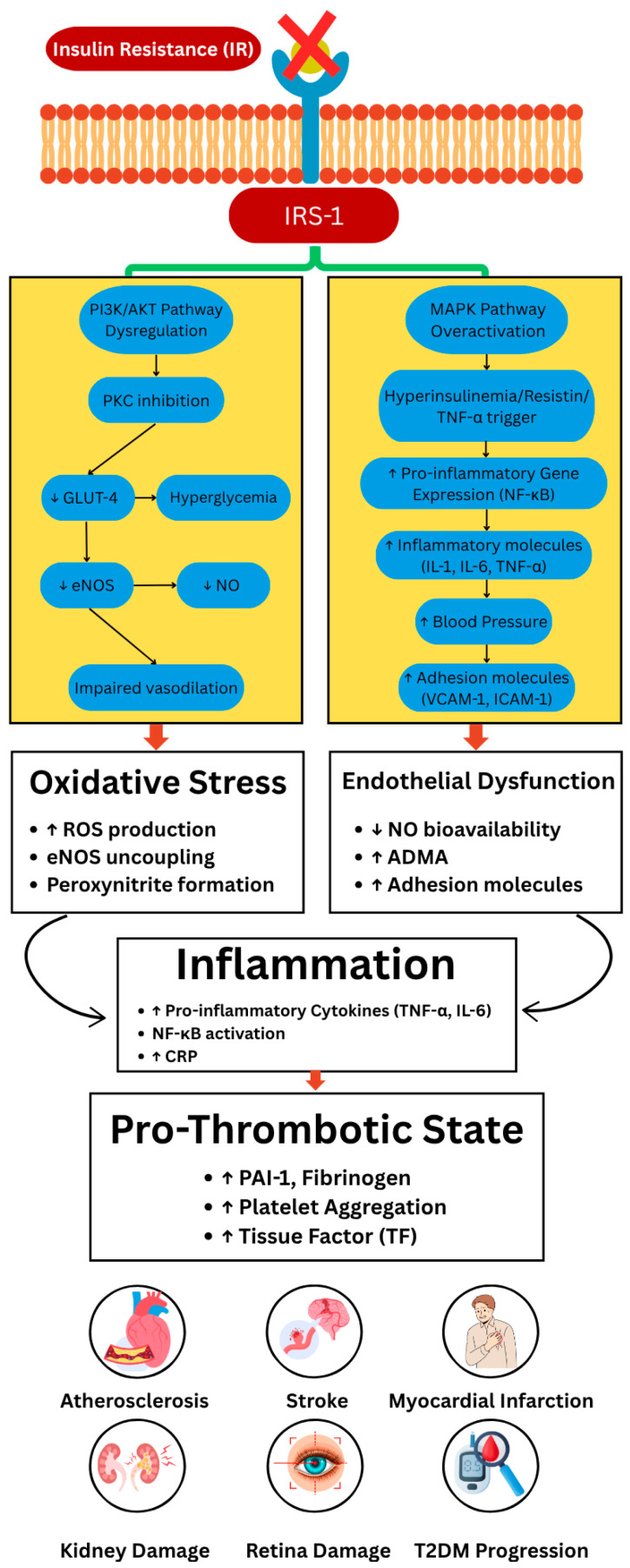
Imbalance between PI3K/AKT and MAPK pathways in insulin resistance contributes to oxidative stress, inflammation, and thrombosis. In insulin resistance, impaired IRS-1 signaling disrupts the PI3K/AKT pathway and favors MAPK overactivation. PI3K/AKT dysregulation leads to reduced GLUT-4 translocation, hyperglycemia, and decreased eNOS and nitric oxide (NO), resulting in impaired vasodilation. Concurrently, MAPK overactivation, driven by hyperinsulinemia, TNF-α, and resistin, enhances NF-κB-mediated expression of inflammatory genes and adhesion molecules. These alterations promote oxidative stress, endothelial dysfunction, and inflammation, which converge into a prothrombotic state and subsequent end-organ damage (PI3K: phosphoinositide 3-kinase; AKT: protein kinase B; MAPK: mitogen-activated protein kinase; IRS-1: insulin receptor substrate-1; GLUT-4: glucose transporter type 4; eNOS: endothelial nitric oxide synthase; NO: nitric oxide; TNF-α: tumor necrosis factor-alpha; NF-κB: nuclear factor kappa-light-chain-enhancer of activated B cells).

IR affects endothelial cells and increases the levels of prothrombotic factors, pro-inflammatory markers, and ROS (reactive oxygen species), which leads to increased levels of intracellular adhesion molecule 1 (ICAM-1) and vascular cell adhesion molecule 1 (VCAM-1) [33]. NO contributes to vascular wall homeostasis by preventing platelet aggregation, inhibiting leukocyte adhesion, and exhibiting anti-inflammatory effects [59]. In cases of IR, the synthesis of insulin-stimulated NO is selectively diminished, and compensatory hyperinsulinemia may trigger the MAPK pathway, resulting in heightened vasoconstriction, inflammation, sodium and water retention, and ultimately increased blood pressure (Figure 1) [60,61]. After insulin binds to the insulin receptor, it activates the PI3K/AKT and MAPK pathways [62]. The PI3K/AKT pathway is essential for insulin action, regulation of glucose uptake, glycogen/lipid synthesis, and mitochondrial activity, and the MAPK pathway is involved in stress and inflammatory responses [63]. Imbalance between the PI3K and MAPK signaling pathways in conditions of insulin resistance leads to disruption of the endothelial PI3K/eNOS and downstream MAPK pathways [64]. Patients with T2DM have increased ROS production and reduced antioxidant defense mechanisms [65]. Increased oxidative stress is a mechanism driving endothelial dysfunction, mainly due to increased superoxide production from the mitochondrial electron transport chain and NAD(P)H-dependent oxidases. Oxidative stress occurs when there is an imbalance between the production of reactive species and the antioxidant defense system [66]. Excess superoxide leads to the formation of peroxynitrites, which interact with the eNOS (endothelial nitric oxide synthase) tetrahydrobiopterin (BH4) factor, causing a decrease in BH4 levels. This deficiency causes eNOS to produce superoxide instead of nitric oxide, contributing to endothelial dysfunction. In addition, hyperglycemia can inhibit dimethylarginine dimethylaminohydrolase (DDAH) and increase the level of asymmetric dimethylarginine (ADMA), which competes with L-arginine and further inhibits nitric oxide production. ROS from hyperglycemia can also impair insulin-stimulated nitric oxide production by affecting the activation of the IRS-1-mediated PI 3-kinase/AKT pathway [67]. Hyperglycemia can independently induce diacylglycerol (DAG) synthesis, which activates PKC (protein kinase C), which in turn inhibits the PI3-kinase/AKT pathway and reduces eNOS phosphorylation and NO production in response to insulin [43]. Hyperglycemia initiates the formation of advanced glycation end products (AGEs). AGEs bind to their receptor RAGE and increase intracellular superoxide production, causing intravascular inflammation [68,69]. Glucotoxicity initiates a slight pro-inflammatory response characterized by increased activation of the pro-inflammatory transcription factor NF-kB. This results in an elevated production of pro-inflammatory cytokines, including TNF-α and various interleukins (IL-1, IL-2, IL-6, and IL-8) [43]. High levels of interleukin 1β (IL-1β), IL-6, and CRP are associated with the prediction of T2DM. Serum concentrations of IL-1 receptor antagonist (IL-1RA) are increased in obesity and prediabetes, indicating a rapid increase in IL-1RA levels before the development of T2DM. Notably, elevated CRP levels are considered the most reliable epidemiological biomarker for cardiovascular disease associated with T2DM. Many of the pro-inflammatory factors found at elevated levels in T2DM patients are IL-1-dependent. High circulating levels of IL-1β, IL-6, and acute phase proteins in T2DM could be due to the activation of innate immune cells due to higher nutrient levels [70]. Fibrosis, a hallmark of inflammation, has been observed in islet tissue from patients with T2DM along with amyloid deposits, which could indicate an inflammatory response. A study demonstrated that human islet amyloid polypeptide (IAPP) induces the secretion of mature IL-1β by bone marrow-derived macrophages, suggesting a possible role for IAPP in islet inflammation [71]. Furthermore, emerging evidence suggests that neutrophil extracellular traps (NETs) contribute to thrombosis in metabolic syndrome and type 2 diabetes [72]. NETs provide a scaffold for platelet adhesion and coagulation factor activation, thereby linking innate immune responses to thrombotic events [72].

## 4. The Effect of Obesity on Thrombosis and Inflammation

Obesity is a major public health issue that is associated with inflammation, CVDs, MetS, and T2DM. Obesity increases the recruitment of macrophages into tissues and their transformation into a pro-inflammatory M1 phenotype. In humans, macrophages constitute 4% of all immune cells in adipose tissue, increasing to 12% in obese individuals. In addition to their phagocytic function, macrophages produce various cytokines that cause chronic inflammation, recruit other inflammatory cells, and produce inflammatory cytokines. M1 macrophages are naturally activated and produce elevated levels of cytokines such as IL-6 and TNF-α, whereas M2 macrophages are intermittently activated and secrete cytokines such as IL-1Rα and IL-10 [73]. Inflammation induced by M1 macrophages in tissues is directly linked to obesity-related IR [74]. MCP-1 produced by preadipocytes leads to increased recruitment of monocytes into adipose tissue, where they differentiate into macrophages. These macrophages secrete cytokines such as TNF-α, which play a role in obesity-induced IR [75]. Inflammatory cytokines stimulate the expression of adhesion molecules such as P-selectin, which mediate endothelial–leukocyte and platelet–leukocyte interactions leading to thrombosis [76]. Fat-resident macrophages gradually increase with the growth of fat mass, with this accumulation being greater in visceral tissue than in subcutaneous adipose tissue [77].

In lean individuals, immune cells function as T helper type 2 (Th2) cells, and IL-33—which is produced primarily by epithelial cells—plays an important role in regulating adipose tissue and metabolism. IL-33 induces the release of IL-5 and IL-13 from innate lymphoid cells and the activation of eosinophils. Eosinophils also produce IL-4, which promotes M2 macrophage polarization and beige fat cell differentiation [78,79]. Immune cells in the adipose tissue of obese individuals function in a pro-inflammatory Th1 mode. Furthermore, key regulators such as TNF-α are secreted by adipocytes and immune cells, which can lead to chronic inflammation and impaired lipid metabolism, leading to obesity-related complications [80]. T cells typically accumulate in adipose tissue alongside macrophages [81]. Generally, CD8+ T cells and TH1 cells promote insulin resistance, while regulatory T cells (TReg) and TH2 cells help mitigate it. TReg cells play a crucial role in maintaining self-tolerance and suppressing potentially autoreactive T cells, thereby preventing the development of autoimmunity in experimental models in both mice and humans. In lean mice, TReg cells make up a notably high proportion, around 50%, of the CD4+ T cell compartment in adipose tissue, which decreases significantly with obesity [53]. Adipose tissue TReg cells express significant amounts of the anti-inflammatory cytokine IL-10, which can suppress adipose tissue inflammation in lean mice [82].

Obesity-related cytokines such as TNF-α and IL-6 stimulate tissue factor expression in endothelial cells and monocytes [83]. Pro-inflammatory cytokines also stimulate the production of coagulation factors such as fibrinogen, FVII, and FVIII, and the fibrinolytic inhibitor PAI-1 in the liver and, by increasing PAI-1 production, they cause platelet activation [84].

Adipokines, or adipocytokines, are cytokines produced by adipose tissue that are involved in the body’s metabolic state, inflammation, obesity, etc. Adipokines include leptin, adiponectin, resistin, IL-6, and TNF-α [85]. Increased adipose tissue releases a variety of cytokines and adipokines, including leptin, TNF-α, adiponectin, and IL-6; moreover, IL-6 increases the production of thrombopoietin in the liver, which can stimulate the proliferation of inflammatory megakaryocytes and megakaryocytes [45]. Leptin may serve as a mediator of inflammation, playing a role in both autoimmune diseases and other inflammatory disorders. Conversely, chronic inflammatory conditions resulting from metabolic, autoimmune, or infectious diseases can induce central leptin resistance, a recognized contributor to obesity. This, in turn, elevates leptin levels and exacerbates the inflammatory state [86]. IL-6 moves through the bloodstream to the liver and rapidly induces the production of various acute-phase proteins such as CRP, serum amyloid A, fibrinogen, haptoglobin, and alpha-1-antichymotrypsin. This adipokine induces hepcidin and increases the maturation of megakaryocytes in the bone marrow, which leads to increased platelet secretion. IL-6, when combined with TGF-b—which induces T helper 17 differentiation—inhibits the formation of Tregs. An imbalance between Th17 and Treg cells can disrupt immune tolerance and contribute to autoimmune disorders and chronic inflammation [87].

A 2020 study conducted by Hana Alzamil showed that serum TNF-α levels were significantly higher in patients with T2DM compared with healthy subjects, and that TNF-α levels had a strong positive correlation with HbA1c. These findings suggested that TNF-α plays an important role in the pathogenesis of T2DM through mechanisms related to the peripheral action of insulin [88]. In obesity, increased TNF-α and free fatty acids (FFAs) lead to chronic activation of JNK (c-Jun *N*-terminal kinase) in the liver, muscle, and adipose tissues. JNK disrupts insulin signaling through phosphorylation of IRS-1 [89]. Binding of TNF-α to TNFR1 activates IKK (IκB kinase), which releases nuclear factor kappa B (NF-Κb) and increases the transcription of inflammatory genes. This inflammation can worsen insulin resistance [88].

**Figure 2:**
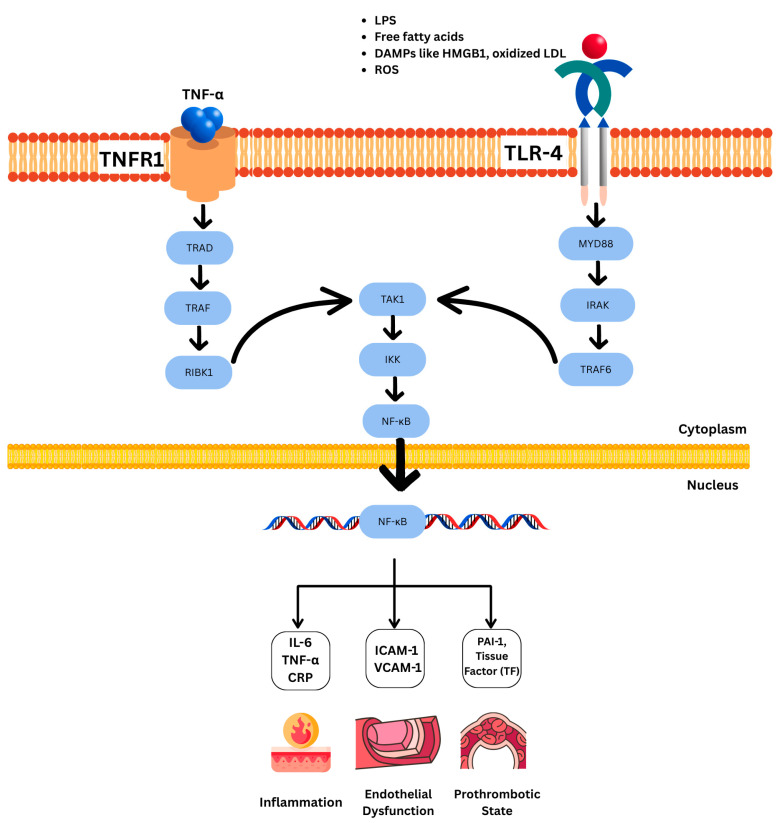
NF-κB pathway activation via TNFR1 and TLR4 in metabolic syndrome. TNF-α and metabolic stimuli (e.g., LPS, ROS, and FFAs) activate NF-κB through TNFR1 and TLR4. Signal transduction converges at TAK1 and IKK, leading to NF-κB nuclear translocation and transcription of inflammatory (IL-6, CRP), adhesive (ICAM-1, VCAM-1), and prothrombotic (PAI-1, TF) genes (TNFR1—TNF Receptor 1; TLR4—Toll-like Receptor 4; LPS—lipopolysaccharide; ROS—reactive oxygen species; FFAs—free fatty acids; CRP—C-reactive protein; ICAM-1—intercellular adhesion molecule-1; VCAM-1—vascular cell adhesion molecule-1; PAI-1—plasminogen activator inhibitor-1; and TF—tissue factor).

TNF-α activates sphingomyelinase and increases ceramide levels. Ceramide activates protein phosphatase 2A (PP2A), which dephosphorylates and inactivates AKT. As AKT is involved in insulin-stimulated glucose uptake and glycogen synthesis, its inhibition leads to IR [90]. TNF-α increases lipolysis and increases free fatty acids in the bloodstream. These fatty acids are elevated in muscle and heart, leading to increased levels of DAG. DAG activates PKC isoforms, which can cause serine phosphorylation of IRS-1 and disrupt insulin signaling [91]. TNF-α has been shown to affect and increase the expression and activity of PTP1B (protein-tyrosine phosphatase 1B). PTP1B dephosphorylates IRS-1 and IRS-2, impairing their ability to transmit insulin signals downstream [92,93]. Additionally, TNF-α enhances tissue factor (TF) expression and promotes thrombotic activity by activating the NF-κB pathway, with the TF promoter directly interacting with NF-κB [94] (Figure 2).

Obesity and insulin resistance are two major components of MetS associated with oxidative stress, suggesting a close relationship between oxidative stress and excessive fat accumulation [95]. In 2020, Zaida Abad-Jiménez et al. showed that patients who underwent gastric bypass surgery had reduced superoxide anion production and increased antioxidant defense in leukocytes, which was associated with improvements in various markers of atherosclerosis [96]. Persistent inflammation in obesity can suppress endogenous antioxidants, such as superoxide dismutase (SOD), catalase (CAT), and glutathione peroxidase (GPx), due to excessive levels of adipokines secreted by fat cells in the body, leading to oxidative stress. In addition, the synthesis of triglycerides and fatty acids, called lipogenesis, is increased in obesity, and lipogenesis initiates the formation of NADPH oxidase through the pentose phosphate signaling mechanism. As a result, fat cells in an obese person’s abdomen can cause acne and inflammation [97].

Retinol binding protein 4 (RBP4) is found in fat cells, macrophages, and the liver, and is higher in people with too much fat. RBP4 activates TLR2/4, which increases certain inflammatory proteins. It also prompts blood vessel cells to produce more inflammation-causing substances, leading to problems with blood flow. Reducing RBP4 levels can help to lower plaque buildup in blood vessels. Resistin is a pro-inflammatory adipokine that interacts with Toll-like Receptor 4 or Adenylyl Cyclase-Associated Protein 1 (CAP1) in endothelial cells and monocytes, triggering pro-inflammatory signals through NFκB and MAPK pathways. It increases the levels of inflammatory molecules such as TNF-α, IL-1, IL-6, and IL-12, and adhesion molecules VCAM1 and ICAM1 via the p38/MAPK pathway and promotes the release of Endothelin-1 (ET-1). Additionally, resistin causes vascular endothelial cell constriction and increases thrombosis risk by lowering eNOS and NO levels while raising free oxygen radicals [98].

Leptin is a hormone that helps control appetite, insulin levels, and obesity by acting on the hypothalamus. Leptin activation of human platelets triggers the JAK2/STAT3 signaling pathway, resulting in enhanced thromboxane production and stimulation of the fibrinogen receptor αIIbβ3, ultimately leading to increased platelet aggregation. Additionally, leptin’s effects on the vascular endothelium involve heightened expression of C-reactive protein and worsening of endothelial dysfunction due to increased protein kinase C-β activity and reduced endothelial nitric oxide production [77]. Leptin has been linked to increased levels of CRP in blood vessels, which is associated with a higher risk of heart disease, and there is a link between leptin and CRP. Marjan Motie and colleagues examined 36 patients for height, weight, waist circumference, body fat percentage, and CRP and leptin levels. They showed that patients with higher body mass index (BMI) had higher CRP and leptin levels [99].

Obesity can lead to increased levels of various intestinal antigens by increasing intestinal permeability, including lipopolysaccharides (LPSs) from Gram-positive bacteria [100]. The microbiota of obese individuals is less diverse. Various studies have shown that dysbiosis in obesity is associated with a reduced percentage of butyrate-producing bacteria, such as *Akkermansia muciniphila, Roseburia*, and *Faecalibacterium prausnitzii*, which may lead to local inflammation and increased intestinal permeability [101]. There is a bidirectional relationship between obesity and dysbiosis, which exacerbates inflammation and oxidative stress in the body. In obese individuals, the ratio of Firmicutes to Bacteroidetes bacteria increases. This imbalance is accompanied by an increase in inflammatory bacteria such as *Escherichia coli* and a decrease in anti-inflammatory bacteria such as *Fecalibacterium prausnitzii,* which itself plays an important role in the exacerbation of inflammatory processes associated with obesity [7].

Circulating LPS activates Toll-like receptors on endothelial cells and platelets, promoting inflammation, endothelial dysfunction, and procoagulant activity [102]. Therefore, LPS may act as an inflammatory stimulus, particularly in visceral fat [103]. This inflammatory environment enhances platelet activation; moreover, aggregation favors fibrin formation and inhibits fibrinolysis, collectively establishing a prothrombotic state [104]. Clinical studies have linked elevated markers of gut permeability and endotoxemia to higher risks of atherosclerosis, CVDs, and IR [102,105].

Obesity has been found to cause localized decreases in PO2, resulting in elevated levels of leptin, IL-6, vascular endothelial growth factor (VEGF), glucose transporter 1 (GLUT1), and PAI-1 [106].

Hypoxia-inducible factor 1 (HIF-1) is higher in the adipose tissue of obese rodents. Genetic deletion of HIF1 has been shown to prevent obesity-associated inflammation and insulin resistance, which could be due to interference with the NF-kB signaling pathway [80].

Obesity is associated with increased concentrations of coagulation inhibitors, such as protein C, AT, and Tissue Factor Pathway Inhibitor (TFPI) [107]. In obese individuals, PAI-1 expression is significantly elevated in visceral adipose tissue, and those with central obesity also display higher circulating levels of PAI-1. Additionally, plasma PAI-1 levels are increased in patients with obesity or MetS [77]. BMI is associated with increased blood clotting after injury. In a study conducted by Kornblith, in 377 trauma patients, obese patients were found to have higher platelet counts, lower D-dimer levels, and higher factor IX activity than normal-weight patients. Thromboelastography results also showed that obese patients had stronger coagulation capacity and higher functional fibrinogen levels at admission. Thus, obese trauma patients show a significantly increased blood clotting compared with normal-weight patients [108]. Table 1 provides a summary of the key coagulation and fibrinolysis markers in MetS. The table highlights the increased levels of fibrinogen, PAI-1, factor VII, and factor VIII, alongside a reduction in fibrinolytic activity, which collectively contribute to the hypercoagulable state observed in MetS.

## 5. The Effect of Hypertension on Thrombosis and Inflammation

Hypertensive patients with MetS have higher 24-h and nocturnal blood pressure levels compared with those without MetS [109]. Components of MetS are independently associated with increased systolic blood pressure variability, and this variability is also linked to organ damage [110]. Furthermore, MetS and its components are associated with alterations in both the structure and function of the heart, including increased right ventricular wall thickness and impaired diastolic function [109].

High blood pressure can increase the risk of thrombosis, which appears to be due to interactions between the renin–angiotensin system and hemostasis [111]. Vascular endothelial cells play a crucial role in linking high blood pressure (hypertension) to thrombosis (blood clot complications). The walls of blood vessels are subjected to two primary mechanical forces: cyclic strain and shear stress. Cyclic strain, caused by internal pressure, leads to vessel expansion and increased wall stress, which raises the expression of eNOS and the production of NO along with elevating levels of superoxide anions. Shear stress is a frictional force that specifically impacts endothelial cells, driven by pressure differences and vessel size. Acute shear stress activates calcium channels, resulting in the release of calcium ions, arachidonic acid, NO, and prostacyclin (PGI2), establishing it as a key regulator of these substances. Sustained shear stress changes the structure of endothelial cells, making them flatter and aligning them with blood flow while promoting cell differentiation and growth. This process activates NF-κB, which boosts the production of various factors, including platelet-derived growth factor-β (PDGF-β), transforming growth factor-β1, t-PA, cyclooxygenase, eNOS, prostacyclin synthase, and TF [112].

When angiotensin I (Ang I), Ang II, or Ang III attaches to the angiotensin type 1 (AT1) receptor on endothelial cell surfaces, it initiates an internal signaling cascade. This process activates several proteins and enzymes, ultimately resulting in the activation of NF-κB and AP-1. These transcription factors then translocate to the cell nucleus, increasing TF gene expression [112,113,114]. Elena Y. Senchenkova and her colleagues demonstrated that hypertension induced by angiotensin II is linked to heightened arterial thrombosis. Studies have shown that Ang I and Ang II can induce an increase in PAI-1 mRNA expression, and this may be attributed to the conversion of these peptides to Ang IV [111]. Kim B.S. et al. showed that hypertensive subjects had higher D-dimer levels compared with controls, and that the levels increased with the severity of hypertension. This indicates that a greater tendency for blood clot formation, as evidenced by elevated levels of the D-dimer, plays a role in the emergence of thromboembolic complications in hypertensive patients [115].

Plasma fibrinogen levels have been shown to be significantly higher in patients with hypertension. This result was consistent with several studies conducted among patients with hypertension [116,117]. Dorfel et al. showed that pre-activation of peripheral blood monocytes in hypertensive patients with angiotensin II can lead to subendothelial infiltration and an increased risk of atherosclerotic complications [118]. As illustrated in Table 2, obesity, hypertension, dyslipidemia, and IR contribute distinctly to both inflammation and hypercoagulability. For instance, increased levels of fibrinogen and PAI-1, which are highlighted in Table 2, are key markers that exacerbate thrombotic risk in MetS.

## 6. Dietary Interventions: Plant-Based and Vegan Diets in MetS Management

The phrase “plant-based diet” encompasses various eating habits characterized by lower intake of animal-derived foods, such as meat and dairy, alongside a greater focus on foods sourced from plants [119]. Both vegan and plant-based (vegetarian) diets exclude meat; however, vegan diets eliminate all animal products, while plant-based diets focus on consuming mostly plant foods and may still include some animal-derived ingredients such as dairy or eggs [120]. When well-balanced, these diets are rich in fiber, vitamins, antioxidants, and healthy carbohydrates [121]. Adequate protein intake on a vegan diet can be achieved by incorporating protein-rich foods such as beans, peas, chickpeas, and products fortified with plant-based proteins [122]. Compared with a plant-based diet, a vegan diet contains almost no cholesterol. Vegan diets are able to provide the lowest calorie density, which is very important for weight loss [120]. The vegan diet that eliminates all animal-derived products has received a lot of media and scientific attention for its possible health benefits, especially in the context of MetS. Current research suggests that a well-structured vegan diet could help improve metabolic disorders. Thus, it might serve as a way to prevent and control MetS [123,124]. A primary advantage of a vegan diet is its association with weight reduction and better body composition. Studies consistently show that individuals following a vegan diet have lower body mass index (BMI) and smaller waist circumference compared to omnivores [125,126]. Moreover, the increased fiber content of the diet is key to obesity reduction, which is a result of better self-control and reduced energy intake. Soluble fiber is crucial for managing blood sugar and insulin levels after meals, which helps improve insulin sensitivity [127,128]. A well-balanced vegan diet may upregulate adiponectin, an anti-inflammatory adipokine with insulin-sensitizing properties, which is often reduced in individuals with MetS [129]. Furthermore, the vegan diet, being free of saturated fats, is optimal for achieving healthy cholesterol and triglyceride levels. It provides a lipid profile characterized by lower levels of low-density lipoprotein cholesterol and triglycerides [130,131]. The impact of vegan diets on blood pressure is a key area of investigation. Several studies have reported that plant-based dietary patterns, including vegan diets, are associated with reductions in both systolic and diastolic blood pressure [132,133]. The likely impact comes from the diet’s high potassium and magnesium, which help widen blood vessels (vasodilation) and lower resistance in those vessels, respectively. Also, polyphenols and other plant compounds (phytochemicals) in these foods have anti-inflammatory and antioxidant effects. In fact, components common in many plant-based dietary patterns, such as those found in the Mediterranean diet, are rich in polyphenols—including naringenin, apigenin, kaempferol, hesperidin, ellagic acid, oleuropein, rosmarinic acid, resveratrol, and quercetin—as well as dietary fiber, monounsaturated fatty acids (e.g., omega-9), polyunsaturated fatty acids (e.g., omega-3), complex carbohydrates, and essential vitamins (A, C, and E) and minerals (including calcium, potassium, and magnesium). This diverse nutrient profile collectively contributes to a reduced risk of metabolic syndrome [134]. Omega-9 fatty acids, particularly oleic acid, possess antioxidant and anti-inflammatory properties that may enhance pancreatic β-cell function, insulin sensitivity, and endothelial health [135]. Oleic acid and polyphenols in olive oil can inhibit the angiotensin-converting enzyme (ACE) pathway, thereby contributing to blood pressure regulation. Vitamins A, C, and E act as antioxidants, which are key factors in insulin resistance, cardiovascular disease, and cancer [136]. By reducing oxidative damage and inflammation, plant-based dietary patterns, exemplified by the Mediterranean diet, support pancreatic β-cell function, enhance insulin sensitivity and secretion, and improve endothelial function. These effects contribute to better blood pressure regulation, lipid metabolism, and overall cardiovascular health [136]. The role of dietary nitrates, abundant in leafy green vegetables, has been increasingly recognized for their ability to enhance endothelial function and reduce blood pressure via nitric oxide-mediated pathways [137]. Emerging evidence also suggests that the gut-derived hormone fibroblast growth factor 21 (FGF21), which is modulated by plant-based nutrient intake, plays a role in improving insulin sensitivity and energy expenditure in individuals with metabolic syndrome [138].

In addition to their beneficial nutrients and bioactive compounds, plant-based diets, including patterns like the Mediterranean diet, are generally low in harmful components, such as saturated and trans fats, excessive omega-6 linoleic acid, cholesterol, simple carbohydrates, sodium, nitrites, and overall fat content [139,140]. Olive oil, a key component emphasized in many healthy dietary patterns, contains polyphenols that help reduce visceral adiposity, insulin resistance, blood pressure, and lipid peroxidation. These compounds also inhibit NF-κB signaling and expression, thereby reducing the production of pro-inflammatory cytokines and contributing to metabolic health [141]. For instance, citrus polyphenols can specifically block the expression of NF-κB, thereby reducing oxidative stress and inflammation in the human body. This reduction in oxidative stress and inflammation can further increase insulin sensitivity, improve fat metabolism, and reduce blood pressure [142]. Moreover, common plant-based food groups such as vegetables, fruits, and spices are particularly rich in polyphenols—bioactive compounds that help counteract oxidative stress and inflammation. In addition to macronutrient composition, the phytochemical diversity in vegan diets, including flavonoids, stilbenes, and lignans, has shown synergistic effects on metabolic regulation and vascular health [143]. Polyphenols in vegan diets, such as epigallocatechin gallate from green tea and curcumin from turmeric, exhibit epigenetic regulatory effects that may suppress inflammatory gene expression implicated in MetS pathophysiology [144]. Plant-based diets are inversely associated with serum levels of advanced glycation end-products, compounds that exacerbate oxidative stress and vascular inflammation in metabolic disorders [145]. As a result, these polyphenols can contribute to increased HDL levels, lower LDL levels, and improved insulin resistance, body mass index, and blood pressure [134]. These actions can further reduce oxidative processes by improving how blood vessel linings work (endothelial function) and lessening oxidative stress [146,147].

These actions can further reduce oxidative processes by improving how blood vessel linings work (endothelial function) and lessening oxidative stress. Recent research indicates that vegan diets modulate the activity of peroxisome proliferator-activated receptors (PPARs), nuclear transcription factors involved in glucose and lipid metabolism, contributing to improved metabolic homeostasis [148]. Long-chain carbohydrates in legumes and whole grains, through fermentation by colonic microbiota, produce short-chain fatty acids like butyrate, which exert systemic anti-inflammatory effects by inhibiting histone deacetylases [149]. Furthermore, vegan diets may modulate expression levels of miRNAs involved in adipogenesis, glucose metabolism, and inflammatory signaling, offering a novel mechanism for dietary intervention in MetS [150]. Lipid metabolism is also favorably influenced by a vegan diet.

The substitution of saturated fats with unsaturated fats, particularly polyunsaturated fatty acids (PUFAs), has been shown to lower LDL-C without adversely affecting high-density lipoprotein cholesterol [151]. Moreover, the inclusion of phytosterols, which compete with cholesterol for absorption in the gut, can further reduce circulating LDL-C levels [152,153]. These lipid-lowering effects are crucial for reducing the risk of atherosclerosis and subsequent cardiovascular events [154]. The replacement of heme iron with non-heme sources from plants may also attenuate oxidative stress, as heme iron has been shown to catalyze free radical formation and lipid peroxidation [155]. Adherence to plant-based eating patterns has been linked to decreased circulating concentrations of lipoprotein (a), an independent cardiovascular risk factor often elevated in patients with MetS [156]. In the context of glycemic control, the vegan diet has demonstrated significant benefits. The high intake of whole grains, legumes, and vegetables, which are rich in complex carbohydrates and fiber, helps to stabilize blood glucose levels and improve insulin sensitivity [157,158]. Clinical trials have reported that individuals following a vegan diet experience greater reductions in fasting blood glucose and HbA1c levels compared with those on conventional diets [159,160]. This is particularly relevant for individuals with T2DM, where improved glycemic control can mitigate the risk of complications [161]. Despite these benefits, it is important to acknowledge potential nutritional deficiencies associated with a vegan diet, particularly in vitamin B12, iron, zinc, and omega-3 fatty acids. These deficiencies can be mitigated through careful dietary planning and supplementation, ensuring that the diet remains nutritionally adequate [162,163]. Dietary alkaloids such as berberine, found in plant sources like Berberis vulgaris, have demonstrated lipid-lowering, hypoglycemic, and anti-inflammatory effects relevant to MetS management [164]. Clinical trials suggest that integrating fermented plant foods into vegan diets, such as kimchi or tempeh, may positively influence metabolic markers through modulation of gut microbial metabolites [165]. Intermittent fasting patterns often adopted alongside plant-based diets may enhance metabolic flexibility and improve mitochondrial biogenesis, further supporting metabolic health in MetS patients [166]. In conclusion, the vegan diet offers a multifaceted approach to mitigating the risk factors associated with metabolic syndrome. Its benefits in weight management, blood pressure regulation, lipid profile improvement, and glycemic control make it a viable dietary strategy for reducing the burden of MetS and its associated comorbidities. However, long-term clinical studies are needed to further elucidate the diet’s impact and to establish comprehensive guidelines for its implementation in diverse populations [167]. Table 3 provides a comparative analysis of metabolic markers before and after adopting a vegan diet, including blood glucose, LDL, HDL, triglycerides, and CRP levels. The data indicate significant improvements in blood glucose and LDL levels following the dietary change. Mahdi Vajdi and colleagues investigated the link between various plant-based diets and MetS in 347 adults with obesity in Iran, aged 20 to 50 years. Their study looked at overall plant-based eating patterns, along with specifically healthy and unhealthy plant-based diets. They found no association between overall or healthy plant-based diets and metabolic syndrome. However, those following an unhealthy plant-based diet were more likely to have high blood sugar and insulin [168]. A meta-analysis was conducted to investigate the effects of plant-based diets on insulin resistance and blood lipids in obese individuals. The results from six studies (seven datasets) showed that a plant-based diet significantly improved insulin resistance, total cholesterol, HDL, and LDL, but had no significant effect on triglycerides [169]. A systematic review and meta-analysis of 13 prospective cohort studies, involving 844,175 participants, investigated the link between vegetarian or vegan diets and the risk of cardiovascular diseases (CVDs), ischemic heart disease, and stroke. This analysis revealed that vegetarian diets were associated with a 15% lower risk of CVDs and a 21% lower risk of ischemic heart disease compared to non-vegetarian diets. Vegan diets also showed a reduced risk of CVDs [170]. Another study compared the food intake and cardiovascular risk markers across three groups: strict vegetarians (no animal products), semi-vegetarians (eggs and dairy, but no meat), and non-vegetarians (meat, dairy, and plants). Strict vegetarians consumed less protein, saturated fat, and cholesterol, but more fiber and polyunsaturated fat. They also showed healthier blood profiles related to clot formation and breakdown, suggesting a lower risk of arterial thrombosis and cardiovascular disease [171].

Isoflavones are polyphenols found primarily in soy and, as phytoestrogens, have beneficial effects on various chronic diseases. Isoflavones have a potential preventive effect on the symptoms of metabolic syndrome by having anti-inflammatory effects and increasing the health of endothelial cells [172]. Menopause is associated with the development of metabolic syndrome, which is largely due to hormonal changes, including estrogen deficiency and increased testosterone activity. Isoflavones, particularly genistein—a soy-derived compound that affects estrogen receptors—have shown beneficial effects on bone health, vascular function, and metabolic markers without adverse effects on the thyroid or reproductive tissues [173]. Soy consumption has been linked to a reduced risk of cardiovascular disease, primarily through improvements in lipid profiles [174]. Soy isoflavones may help control obesity by regulating lipid metabolism through gene expression changes, as shown in animal studies [175,176]. The FDA recognizes that consuming 25 g of soy protein daily can help lower total and LDL cholesterol, supporting heart disease prevention [177]. Asal Neshatbini Tehrani et al. (2024) showed that soy isoflavones can significantly reduce triglycerides, LDL, and total cholesterol in patients with non-alcoholic fatty liver disease [178]. A study showed that greater adherence to a Mediterranean diet was associated with reduced fibrinogen and non-esterified fatty acids. The Mediterranean diet, especially when enriched with olive oil or nuts, improves cardiovascular risk markers in high-risk individuals [179].

Vegan diets have been shown to contribute to body weight loss, particularly visceral fat reduction, and glucose homeostasis [121]. A vegan diet leads to improved intake of macronutrients, fiber, and cholesterol, thereby creating a beneficial dietary pattern in the prevention and treatment of obesity [180]. This effect is likely attributed to compounds found in plant-based foods such as fibers, chlorogenic acids, antioxidants, plant proteins, and omega-6 fatty acids. These components work by promoting satiety and modulating the gut microbiota, reducing inflammation and decreasing oxidative stress [121].

## 7. The Effect of a Vegan Diet on Gut Microbiota and Metabolic Syndrome

The human gut microbiota plays a central role in maintaining metabolic health. Its composition and functional output are strongly influenced by dietary patterns. Vegan diets, which exclude all animal products and emphasize fiber-rich plant-based foods, have been associated with beneficial shifts in gut microbiota composition and improvements in metabolic parameters related to MetS [181,182]. A growing body of evidence suggests that individuals following a vegan diet exhibit a distinct microbial profile compared with omnivores. Specifically, vegans show increased abundance of *Prevotella* species, which are adept at fermenting complex polysaccharides, and a lower prevalence of *Bacteroides* and other pro-inflammatory bacteria associated with Western dietary patterns [183,184]. This shift is attributed to the high intake of dietary fiber, polyphenols, and resistant starches, which serve as substrates for short-chain fatty acid (SCFA) production, particularly butyrate, propionate, and acetate. These SCFAs exert anti-inflammatory effects, improve gut barrier function, and modulate glucose and lipid metabolism [185]. Metabolic syndrome—a cluster of conditions including central obesity, dyslipidemia, hypertension, and IR—is closely linked to systemic inflammation and gut dysbiosis. A vegan diet may attenuate these risk factors via modulation of gut microbial ecology. In a 2020 randomized controlled trial, Kahleova et al. demonstrated that a low-fat vegan diet led to significant weight loss, improved insulin sensitivity, and reduced hepatic fat in overweight individuals, with concurrent alterations in gut microbiota composition and function [122]. Similarly, a cross-sectional analysis from the American Gut Project indicated that plant-based diets correlated with greater microbial diversity, a marker of gut health, and lower levels of trimethylamine-N-oxide (TMAO), a metabolite derived from animal protein that is implicated in cardiometabolic disease [186]. Notably, vegan diets also reduce the relative abundance of endotoxin-producing bacteria such as *Bilophila wadsworthia* and *Escherichia coli*, whose lipopolysaccharides (LPSs) contribute to metabolic endotoxemia and chronic low-grade inflammation, which is a driver of IR and atherogenesis [187]. The decreased translocation of LPSs into systemic circulation, likely due to improved gut barrier integrity under a high-fiber regimen, underscores one mechanistic pathway by which vegan diets mitigate metabolic dysfunction [188]. However, long-term adherence to a poorly planned vegan diet may risk deficiencies in vitamin B12, iron, and omega-3 fatty acids, potentially affecting host–microbe interactions and metabolic outcomes. Therefore, nutritional adequacy and diversity within vegan diets are crucial for maximizing their protective effects [189]. A combined effect of prospective studies and clinical trials conducted by Christina-Maria Kastorini, MSc, and colleagues showed that adherence to the Mediterranean diet is associated with a reduced risk of MS [190].

## 8. Current Research Limitations and Future Directions

While the evidence supporting vegan and plant-based diets in the management of MetS is compelling, it should be noted that there are limitations. A number of studies have focused on a subset of individuals, such as those attending a specific hospital, which limits the generalizability of findings to other populations. Data such as MPV are measured using a specific analyzer and, therefore, the results may not be directly comparable with those obtained with other hematology analyzers, leading to measurement variability across laboratories.

Another limitation that must be considered is the heterogeneity of plant-based diets. The terms “vegan” and “vegetarian” encompass a wide range of dietary patterns that can have significantly different nutritional compositions. This diversity makes it difficult to conclude. Consequently, the beneficial effects attributed to plant-based diets in the scientific literature require more precise classification and assessment of diet quality in future research. Most of the available studies are from Western or high-income countries, which limits the comparison of results to other populations with different dietary patterns. Furthermore, the group of studies is heterogeneous in terms of design and method of diagnosing metabolic syndrome, which limits comparison and complicates combined interpretation. In particular, most studies have examined individual components (especially obesity) rather than metabolic syndrome, which limits the ability to draw conclusions about metabolic syndrome. Additionally, many of the trials had short follow-up periods, which limits the ability to assess the long-term effects of plant-based or Mediterranean dietary patterns on inflammation, weight, and insulin resistance. Therefore, long-term randomized controlled trials are needed to assess their impact on inflammation, thrombosis, and cardiovascular clinical outcomes. In addition, personalized nutritional approaches combining genetic and microbiome profiles could optimize dietary recommendations for patients. Addressing nutritional deficiencies through supplementation and studying the role of the gut microbiota will further refine dietary guidelines. Finally, public health efforts should focus on increasing the accessibility, cultural relevance, and adherence to plant-based diets in diverse populations.

## 9. Discussion

This comprehensive review highlights the pathophysiology and the central role of metabolic syndrome in the development of chronic inflammation and thrombosis—two closely related processes in cardiovascular morbidity and mortality. The analysis presented in Table 2 shows how the components of MetS contribute to the pathogenesis of inflammation and thrombosis. Obesity induces chronic inflammation primarily through the secretion of pro-inflammatory cytokines, such as IL-6 and TNF-α, and the accumulation of macrophages in adipose tissue. This resulting inflammation increases PAI-1 expression and platelet aggregation, impairing fibrinolysis. In a study conducted by Anna Tylutka et al. in 2023, it was shown that patients with metabolic syndrome have higher levels of inflammatory markers such as TNF-α and High-Mobility Group Box 1 (HMGB1) protein, as well as high body fat and insulin resistance. This study identified lipid aggregation product (LAP), TNF-α, and HMGB1 as useful predictors of metabolic syndrome, with LAP showing the highest diagnostic value. They showed that chronic low-grade inflammation associated with increased visceral fat plays a key role in the development of metabolic syndrome in the elderly, and the combination of anthropometric and inflammatory markers may improve early diagnosis and management [191]. While some studies suggest that the relationship between MetS and inflammatory markers is primarily driven by its components, there is a hypothesis that genetics can also simultaneously influence multiple metabolic traits and inflammatory markers, suggesting a complex pleiotropic network [192]. Hypertension exacerbates oxidative stress and endothelial dysfunction, further stimulating pro-inflammatory pathways such as NF-κB, thereby increasing fibrinogen levels and thrombotic activity. Dyslipidemia contributes to inflammation through oxidative modification of LDL particles, which activates immune responses and accelerates atherosclerotic plaque formation, and exacerbates the potential for thrombosis by increasing platelet activity and coagulation factor levels. Through activation of macrophages and increased cytokine production, IR causes inflammation, impairs endothelial nitric oxide synthesis, increases ROS production, and increases platelet reactivity and aggregation. Ultimately, these pathological mechanisms in metabolic syndrome and their role in the development of thrombosis and coagulation emphasize the importance of strategies such as dietary modification to break this cycle. While vegan diets provide anti-inflammatory and antioxidant benefits—evidenced by reductions in CRP, IL-6, LDL cholesterol, and blood glucose levels—they may also lead to deficiencies in critical nutrients such as vitamin B12, DHA, EPA, and iron, which are essential for brain function. A systematic review found that obesity, particularly abdominal (visceral) obesity, is consistently associated with the development and severity of metabolic syndrome. In this study, other components of MetS, such as diabetes and hypertension, showed more variable associations. Interestingly, while inflammation is known to be involved in thrombosis and vascular injury, inflammatory markers such as CRP and D-dimer were not significantly different between patients with and without post-thrombotic syndrome, suggesting that local, rather than systemic, inflammation may be key. This review found that visceral fat and its associated inflammatory environment—not metabolic syndrome as a whole—are the most important factors in the development of MetS [193].

Although a vegan diet may contribute to the reduction in MetS disorders through various mechanisms presented in this article, it can also lead to nutritional deficiencies of nutrients such as B12, iron, zinc, and omega-3 fatty acids. As Roman Pawlak et al. have shown, a vegetarian diet causes a decrease in vitamin B12 (Table 4) [30].

Vitamin B12 (Cobalamin) This is the most critical nutrient for vegans, as it is primarily found in animal-derived products and is largely absent in plant foods [194,195,196]. Deficiency can lead to severe neurological and hematological symptoms, particularly in infants if diagnosis or treatment is delayed [194,195,197,198]. The risk of cobalamin deficiency among vegans is strongly linked to the non-use or irregular use of supplements, rather than the duration of adherence to a vegan diet [199]. Studies indicate that regular supplement users can maintain vitamin B12 levels comparable with non-vegans [199]. However, the proportion of vitamin B12 absorbed from large oral doses in supplements is considerably lower than from food, and general Recommended Dietary Intake (RDI)-level supplementation may be inadequate due to these absorption limitations [198]. Multi-nutrient supplements may also contain other nutrients that can inactivate vitamin B12 [198]. Therefore, consistent and reliable supplementation with vitamin B12, often through fortified foods or single-nutrient dietary supplements, is considered essential for individuals following a vegan diet [194,195,196,197,198]. Daily doses of at least 25 micrograms (mcg) or weekly doses of 1000 mcg (preferably taken on an empty stomach to maximize passive absorption) may be required to meet recommendations, with higher doses needed during pregnancy and lactation [198]. Regular monitoring of B12 levels is also advised to ensure adequacy and guide personalized supplementation [194,195,197,199].

Vegans typically consume fewer *n*-3 fatty acids and have lower serum levels of Omega-3 Fatty Acids (EPA and DHA) due to the absence of marine-sourced fats in their diet [196,200]. While alpha-linolenic acid (ALA) from plant sources (such as flaxseeds, walnuts, and chia seeds) can be converted to EPA and DHA, their conversion efficiency in humans is limited, especially for DHA [196]. Therefore, microalgae oil, which is rich in DHA (and EPA), is recommended as a useful supplement for vegans and vegetarians to ensure adequate intake of these crucial fatty acids for cardiovascular and neurological health [196,197].

Beyond these factors, a well-planned plant-based diet should also consider other potentially critical nutrients such as iron, zinc, calcium, iodine, riboflavin, and vitamin D, which may require strategic food choices, fortification, or additional supplementation depending on individual needs and dietary composition [194,195,196,197]. For instance, vegans, vegetarians, and pescatarians in some regions may be at risk of iodine deficiency, as food sources alone might be insufficient [201]. Consulting a qualified nutrition professional can help tailor dietary plans and supplementation protocols to individual requirements, ensuring optimal health outcomes [194,197].

This review also demonstrated that vegan diets improve microbial diversity and reduce LPS-related endotoxemia. In a 2018 study, Daniel McDonald and colleagues showed that vegan dietary diversity can also lead to greater diversity in the gut microbiota. Importantly, they showed that plant species diversity, rather than dietary labels such as “vegan,” was a stronger predictor of gut microbial diversity. This supports our contention that dietary diversity—particularly fiber-rich plant foods—is key in modulating gut health and reducing LPS-induced endotoxemia. A 2025 systematic review and meta-analysis by Khodarahmi et al. examined 60 randomized controlled trials including 5511 adults to assess the effects of low-carbohydrate diets on CRP, a key marker of chronic low-grade inflammation. The study found that low-carbohydrate diets resulted in modest overall reductions in CRP levels. Subgroup analyses showed more pronounced CRP reductions in individuals with higher baseline CRP levels (>4.5 mg/L), obesity, younger age, and those participating in longer-term interventions. The findings suggest that while low-carbohydrate diets may help reduce inflammation, their effectiveness varies depending on individual characteristics and study design [202].

Shifting towards plant-based dietary patterns is crucial for addressing environmental sustainability and improving public health outcomes, often proving more cost-effective and nutritionally beneficial than meat-based options [203,204]. These interventions, when designed with cultural relevance, offer significant societal value by promoting healthy weight loss at a lower cost [205,206]. Cost-effectiveness analyses are vital for evaluating the feasibility of large-scale implementation, especially for diverse populations [205,207]. Promoting plant-based diets as an affordable choice can broaden their acceptance, benefiting lower-income individuals and mitigating food insecurity [208]. The food service sector is key in facilitating these transitions, improving nutrient intake, reducing environmental impact, and lowering costs [203].

Given the global increase in obesity and MetS, particularly in urban and industrialized populations, plant-based diets with appropriate nutritional planning could serve as a preventive public health strategy. This dietary pattern provides anti-inflammatory and antithrombotic benefits that are important for reducing the burden of cardiovascular disease. Altogether, this review highlights that well-planned vegan diets, rich in diverse whole plant foods, offer a powerful, multifactorial tool for disrupting the inflammatory–thrombotic cycle central to MetS and cardiovascular risk.

## 10. Conclusions

This review demonstrated that MetS is not only a metabolic disorder but also a driver of thrombosis and inflammation, increasing the risk of CVD and T2DM. Central obesity, insulin resistance, dyslipidemia, and hypertension create a pathological cycle involving oxidative stress, endothelial dysfunction, and prothrombotic states that collectively pose major challenges to public health.

Vegan diets may play a role in reducing the burden of MetS by improving insulin sensitivity, lipid profiles, blood pressure, and inflammatory markers. Additional benefits on the gut microbiota and endothelial health further enhance their therapeutic potential.

Overall, the implementation of plant-based nutrition in preventive and therapeutic strategies could significantly improve metabolic and vascular outcomes; therefore, further clinical research is warranted.

## Figures and Tables

**Table 1:** Key coagulation and fibrinolysis markers in metabolic syndrome [29,54,84,108].

	Marker	Changes in MetS	Impact
Coagulation Factors	Fibrinogen	Increased levels	Enhances blood viscosity and promotes clot formation
	Factor VII	Higher activity	Accelerates thrombin generation, contributing to coagulation
	Factor VIII	Elevated levels	Promotes the clotting cascade and thrombin generation
	Platelet Activity	Enhanced aggregation	Increases thrombus formation and vascular occlusion
Fibrinolysis Factors	PAI-1	Elevated expression	Inhibits plasminogen activators, reducing fibrinolysis and increasing clot persistence
	tPA (Tissue Plasminogen Activator)	Reduced activity	Impaired fibrin breakdown, leading to reduced fibrinolysis
	D-dimer	Increased concentration	Indicates heightened thrombotic activity and fibrin degradation

**Table 2:** Comparative contributions of metabolic syndrome components (obesity, hypertension, dyslipidemia, and insulin resistance) to inflammation and thrombosis. The information was compiled and synthesized from various scientific studies reviewed in this article.

Factor	Contribution to Inflammation	Contribution to Thrombosis
Obesity	Increases pro-inflammatory cytokines such as IL-6 and TNF-α	Enhances PAI-1 production, impairing fibrinolysis
	Accumulation of macrophages in adipose tissue	Raises levels of fibrinogen and coagulation factors
	Hypoxia in adipose tissue triggers inflammatory responses	Promotes platelet aggregation through leptin
	Gut dysbiosis amplifies local inflammation	
Hypertension	Endothelial activation and oxidative stress	Elevates fibrinogen levels
	Increased activity of NF-kB pathways	Raises PAI-1 expression, reducing clot breakdown
	Not directly addressed	Induces D-dimer elevation, reflecting higher thrombotic activity
Dyslipidemia	Oxidative modification of LDL triggers inflammatory cascades	Enhances platelet hyperactivity
	Accelerates atherosclerotic plaque formation	Increases coagulation via elevated clotting factors
Insulin Resistance	Chronic low-grade inflammation due to macrophage activation	Promotes hypercoagulability via increased fibrinogen and prothrombotic factors
	Reduces endothelial nitric oxide, impairing vascular relaxation	Alters platelet reactivity, increasing aggregation risk
	Enhances ROS production	Not directly addressed

**Table 3:** Comparison of key metabolic syndrome markers in individuals following omnivorous vs. vegan diets. The data are summarized from clinical trials and observational studies identified in this review.

Marker	Omnivorous Diet	Vegan Diet
Blood Glucose	Higher fasting glucose levels due to refined carbohydrates and saturated fats	Lower fasting glucose levels; improved insulin sensitivity from high fiber intake
LDL Cholesterol	Elevated LDL levels from saturated fats and cholesterol in animal products	Reduced LDL levels due to the absence of animal fats and the inclusion of phytosterols
HDL Cholesterol	Moderate HDL levels, influenced by dietary fats	Slightly lower HDL levels, but balanced by improved overall lipid profile
Triglycerides	Higher triglycerides linked to processed foods and simple sugars	Lower triglycerides due to high fiber and unsaturated fats
Inflammatory Markers	Elevated CRP and IL-6 levels from pro-inflammatory animal fats	Reduced CRP and IL-6 levels due to anti-inflammatory phytonutrients and omega-3s

**Table 4:** Comparison of key nutrient levels in vegan vs. omnivorous diets and recommended strategies for vegans to ensure nutritional adequacy. The information is based on current nutritional guidelines and research findings in this review.

Nutrient	Levels in a Vegan Diet	Levels in an Omnivorous Diet	Possible Solutions for Vegans
Vitamin B12	Low, as it is primarily found in animal-derived products	Sufficient, due to sources such as meat, eggs, and dairy	Fortified foods (e.g., cereals and plant-based milk) and B12 supplements
Iron	Non-heme iron (less bioavailable)	Heme iron (more bioavailable from red meat)	Include iron-rich plant foods (e.g., lentils, tofu, and spinach) with vitamin C to enhance absorption
Omega-3	Low levels of long-chain omega-3 fatty acids (EPA and DHA)	Higher levels from fish and seafood	Consume plant sources (e.g., flaxseeds, chia seeds, and walnuts) or algae-based omega-3 supplements
Zinc	Moderate but less bioavailable from plant sources	Sufficient, as animal products are rich in bioavailable zinc	Include zinc-rich foods (e.g., beans, nuts, and seeds) and consider zinc supplements if needed

## Data Availability

We used PubMed, SCOPUS, and ScienceDirect databases to screen articles for this article. No data are reported in this article.

## Abbreviations

The following abbreviations are used in this manuscript:

MetS	Metabolic syndrome
CVDs	Cardiovascular diseases
CRP	C-reactive protein
hs-CRP	High-sensitivity C-reactive protein
IR	Insulin resistance
MPV	Mean platelet volume
FBS	Fasting blood glucose
vWF	von Willebrand factor
T2DM	Type 2 diabetes
PAI-1	Plasminogen activator inhibitor-1
ICAM-1	Intracellular adhesion molecule 1
VCAM-1	Vascular cell adhesion molecule
IL-1RA	IL-1 receptor antagonist
IAPP	Islet amyloid polypeptide
BMI	Body mass index
MCP-1	Protein monocyte chemokine-1
ROS	Reactive oxygen species
PP2A	Protein phosphatase 2A
Treg	Regulatory T cells
PKC	Protein kinase C
AGEs	Advanced glycation end products
TLR4	Toll-like receptor 4
LPS	Lipopolysaccharide
HIF-1	Hypoxia-inducible factor 1
tPA	Tissue plasminogen activator
uPA	Urokinase-type plasminogen activator
GLUT1	Glucose transporter 1
Th2	T helper type 2
PGI2	Prostacyclin
PI3K	Phosphatidylinositol 3-kinase
MAPK	Mitogen-activated protein kinase
IRS	Insulin receptor substrates
BH4	Tetrahydrobiopterin
DAG	Diacylglycerol
RBP4	Retinol binding protein 4
NF-Κb	Nuclear factor kappa B
TF	Tissue factor
AT1	Angiotensin type 1 receptor
Ang	Angiotensin
IKK	IκB kinase
JNK	c-Jun *N*-terminal kinase
GP	Glycoprotein
MC4R	Melanocortin 4 receptor
TCF7L2	Transcription factor 7-like 2
ADIPOQ	Adiponectin gene
GWAS	Genome-wide association studies
FBG	Fasting blood glucose
MCP1	Monocyte chemokine 1
DDAH	Dimethylarginine dimethylaminohydrolase
ADMA	Asymmetric dimethylarginine
NET	Neutrophil extracellular trap
SOD	Superoxide dismutase
CAT	Catalase
GPx	Peroxidase
ET-1	Endothelin-1
PTP1B	Protein-tyrosine phosphatase 1B
TFPI	Tissue factor pathway inhibitor
LDL-C	Low-density lipoprotein cholesterol
VEGF	Vascular endothelial growth factor
PDGF-β	Platelet-derived growth factor-β
PUFAs	Poly unsaturated fatty acids
SCFA	Short-chain fatty acid
TMAO	Trimethylamine-N-oxide
ALA	α-linolenic acid
CAP1	Cyclase-associated protein 1
HMGB1	High-mobility group box 1 protein
